# The Matron's Department

**Published:** 1905-08-26

**Authors:** 


### THE MATRON'S DEPARTMENT.

### III. NURSES AND BEDS.

The first and most anxious subject for the newly-appointed matron to consider is the nursing staff allotted her relatively to the number of beds. Varied experience will here prove her weightiest ally. To the matron trained and employed in but one hospital it will always be a matter of some difficulty to believe that the work can be got through with anything short of the proportion of nurses to which she has been accustomed. But it is quite within the bounds of possibility that she will find but two pairs of hands available for every three at her old training school. I assume, for the sake of argument, that she has been trained at one of the great London hospitals with medical schools. The facts relating to the size of the staff in hospitals throughout the country are extremely obscure. The disproportion revealed in the comparative tables of "Burdett's Hospitals and Charities, 1904," pp. 120, 121, 127, relating to the nursing staff in various institutions is even more remarkable when closely examined, and has never been explained. We quote some of the figures:—

| Name of Institution. | Daily Average of Occupied Beds. | Total Nursing Staff. | Nursing Staff Outside Wards. | Average Number of Beds to each Nurse, Reckoning Ward Staff only. |
|---|---|---|---|---|
| Hospitals with Medical Schools. | | | | |
| The London ... ... ... | 676 | 395 | 57 | 2 |
| Guy's ... ... ... ... | 499 | 261 | 28 | 2·1 |
| St. Bartholomew's ... ... | 535 | 241 | 13 | 2·3 |
| St. Thomas's ... ... ... | 442 | 165 | 12 | 2·8 |
| St. George's ... ... ... | 269 | 144 | 10 | 2 |
| The Middlesex... ... ... | 312 | 138 | 10 | 2·4 |
| St. Mary's ... ... ... | 255 | 100 | 12 | 2·9 |
| King's College ... ... ... | 184 | 81 | 6 | 2·4 |
| University ... ... ... | 166 | 79 | 13 | 2·5 |
| Charing Cross ... ... ... | 137 | 61 | 5 | 2·4 |
| The Royal Free ... ... | 145 | 50 | 4 | 3·1 |
| Birmingham General ... ... | 289 | 101 | 11 | 3·2 |
| Newcastle Royal ... ... | 266 | 74 | 7 | 4 |
| Sheffield Royal ... ... | 208 | 67 | 5 | 3·3 |
| Bristol General ... ... | 164 | 88 | 11 | 2·1 |
| Addenbrooke's, Cambridge ... | 99 | 46 | 4 | 2·3 |
| Radcliffe, Oxford ... ... | 116 | 38 | 5 | 3·5 |
| Edinburgh Royal ... ... | 743 | 226 | 12 | 3·4 |
| Glasgow Royal ... ... | 544 | 158 | 17 | 3·8 |
| Glasgow Western ... ... | 441 | 151 | 9 | 3·1 |
| Belfast Royal Victoria ... | 158 | 66 | 8 | 2·7 |
| General Hospitals without Medical Schools. | | | | |
| Seaman's ... ... ... | 235 | 46 | 5 | 5·3 |
| Great Northern Central ... | 144 | 44 | 5 | 3·7 |
| Norfolk and Norwich ... ... | 169 | 53 | 5 | 3·5 |
| Bradford ... ... ... ... | 185 | 55 | 6 | 3·7 |
| Royal Devon and Exeter ... | 150 | 81 | 6 | 2 |
| Sunderland ... ... ... | 187 | 43 | 5 | 4·9 |
| Sussex County... ... ... | 176 | 62 | 7 | 3·2 |
| Hull Royal ... ... ... | 159 | 43 | 7 | 4·4 |
| Derby Royal ... ... ... | 161 | 56 | 9 | 3·3 |
| Reading Royal, Berks... ... | 132 | 48 | 4 | 3 |

The average number of beds to each nurse has been ascertained by deducting from the staff all those who are mainly employed outside the wards in the out-patient department, the receiving-room, in massage or electricity, and in the office and home departments. The number of occupied beds has been divided by the total remaining staff and the quotient gives, roughly speaking, the average number of patients to each nurse. We say roughly speaking, for this must necessarily be an artificial method of stating the case, although it is the only one which enables the nursing staff of one hospital to be compared with that of another. The nurses must be divided again into day and night staff, some reserved for special duty, and allowance made for relieving those on short and long leave before the real proportion of work, which falls on an average to each member of the staff, can be estimated. And into these calculations it is impossible to enter with a large number of institutions varying in size and in organisation.

But regarded merely as a basis for comparison, the method employed in "Burdett's Hospitals and Charities" is excellent. The true significance of the figures is apparent on a moment's consideration. The difference between an average of two and three beds apiece for the staff works out at a difference of 17 nurses for every 100 beds. The fine gradations can only be ascertained by the use of decimals. The difference between 2 and 2·1 beds apiece is almost exactly a difference of two nurses on every 100 beds. In the case of a hospital with several hundred beds the difference between an average of 2 beds apiece to the ward staff, and say 3·5, or 4 beds, would represent an increase in the latter case of over a hundred nurses.

Various causes conduce to the diversity we have observed, but the cause which may seem the most obvious—namely a distinction in the standard of the nursing, must on mature consideration be altogether rejected. No one who has had any experience of the best hospital work in London and the provinces can consent to the suggestion that the standard of nursing depends to any considerable extent upon the size of the staff.

The construction of the building is one great factor in determining the number of the nursing staff. Large wards of about equal size, containing from 25 to 30 beds, afford fewer difficulties in apportioning the staff than the irregularly built and smaller wards of the older hospitals. It is true that in old hospitals one sister has often the superintendence of two wards which are practically separate although classed as one. But in this case and especially if the rooms which make up the ward are on separate floors, as was the case in the old University College Hospital building, the sister's time is continually wasted, the strain upon her is doubled, and she must have increased help if the work is to be got through at all. The tendency of modern hospital architecture is to reduce labour as far as possible and we find accordingly that among the great London hospitals, the oldest buildings—namely, the London, St. Bartholomew's, Guy's, and St. George's, are those in which the nursing staff reaches its largest proportions.

Another factor which influences the size of the nursing staff is the amount of teaching which is undertaken. In some of the great training schools of the metropolis and the provinces the number of first-year probationers among the nursing staff reaches a total of almost 50 per cent.

In contrasting the staff at her disposal in a medium-sized hospital with that of her old training school, the matron must not omit to take into consideration the quality of the material at her disposition. The cluster of new probationers which goes to swell the size of the staff means a good deal of additional work for all connected with them in their first few weeks or months of hospital life, and from the point of view of actual nursing they are almost a negligible quantity. But when due allowance has been made for difficulties of construction, for the demands of incessant teaching, and for the additional strain of work conducted at the high pressure, which is often a direct consequence of enthusiastic medical schools, there remains still, for the administrative powers of the matron, a large field. In assigning the proper complement of nurses to each ward, in arranging the time-table so that while undue strain on individuals is avoided each nurse may be working up to the full extent of her powers, in dovetailing with precision and economy of labour the services of extra nurses required for leave and holidays, there is scope for ability and certainly need for unceasing vigilance if the number of the staff is to be kept low. We propose, in an ensuing article, to give some examples of the manner in which these practical difficulties are being successfully overcome.

